# Pharmacological mechanism and therapeutic efficacy of Icariside II in the treatment of acute ischemic stroke: a systematic review and network pharmacological analysis

**DOI:** 10.1186/s12906-022-03732-9

**Published:** 2022-09-30

**Authors:** Xu Wang, Jinjian Li, Lifang Liu, Jun-Ming Kan, Ping Niu, Zi-Qiao Yu, Chunyu Ma, Fuxiang Dong, Mo-Xuan Han, Jinhua Li, De-xi Zhao

**Affiliations:** 1 College of Traditional Chinese Medicine, Changchun University of Chinese Medicine, Changchun, Jilin, 130117 China; 2 School of Public Health, Jilin University, Changchun, Jilin, 130021 China; 3 Department of Encephalopathy, Hospital of Changchun University of Chinese Medicine, Changchun, Jilin, 130021 China

**Keywords:** Phosphodiesterase 5 inhibitors, cGMP-PKG signaling pathway, Icariside II, Epimedii, Acute ischemic stroke

## Abstract

**Background and objective:**

Epimedii has long been used as a traditional medicine in Asia for the treatment of various common diseases, including Alzheimer's disease, cancer, erectile dysfunction, and stroke. Studies have reported the ameliorative effects of Icariside II (ICS II), a major metabolite of Epimedii, on acute ischemic stroke (AIS) in animal models. Based on network pharmacology, molecular docking, and molecular dynamics (MD) simulations, we conducted a systematic review to evaluate the effects and neuroprotective mechanisms of ICS II on AIS.

**Methods:**

First, we have searched 6 databases using studies with ICS II treatment on AIS animal models to explore the efficacy of ICS II on AIS in preclinical studies. The literature retrieval time ended on March 8, 2022 (Systematic Review Registration ID: CRD42022306291). There were no restrictions on the language of the search strategy. Systematic review follows the Patient, Intervention, Comparison and Outcome (PICO) methodology and framework. SYCLE's RoB tool was used to evaluate the the risk of bias. In network pharmacology, AIS-related genes were identified and the target-pathway network was constructed. Then, these targets were used in the enrichments of Kyoto Encyclopedia of Genes and Genomes (KEGG) pathways and gene ontology (GO). Molecular docking and MD simulation were finally employed between ICS II and the potential target genes.

**Results:**

Twelve publications were included describing outcomes of 1993 animals. The literature details, animal strains, induction models, doses administered, duration of administration, and outcome measures were extracted from the 12 included studies. ICS II has a good protective effect against AIS. Most of the studies in this systematic review had the appropriate methodological quality, but some did not clearly state the controlling for bias of potential study. Network pharmacology identified 246 targets with SRC, CTNNB1, HSP90AA1, MAPK1, and RELA as the core target proteins. Besides, 215 potential pathways of ICS II were identified, such as PI3K-Akt, MAPK, and cGMP-PKG signaling pathway. GO enrichment analysis showed that ICS II was significantly enriched in subsequent regulation such as MAPK cascade. Molecular docking and MD simulations showed that ICS II can closely bind with important targets.

**Conclusions:**

ICS II is a promising drug in the treatment of AIS. However, this systematic review reveals key knowledge gaps (i.e., the protective role of ICS II in women) that ICS II must address before it can be used for the treatment of human AIS. Our study shows that ICS II plays a protective role in AIS through multi-target and multi-pathway characteristics, providing ideas for the development of drugs for the treatment of AIS.

**Supplementary Information:**

The online version contains supplementary material available at 10.1186/s12906-022-03732-9.

## Introduction

Stroke is a common disease with a high incidence, high mortality and high disability rate in the world. In the United States, the incidence of stroke was 115 per 100,000 people, the mortality was 29 per 100,000 people, and there was a total of 1,536 strokes per 100,000 people, with a total of 7.8 million strokes and the highest burden in terms of disability-adjusted life years (DALYs) [1]. In the 28 countries of European Union (EU28), 7.3 million strokes are the most common source of DALYs [2]. China faces the world's largest challenge with an incidence of 246.8 strokes per 100,000 people and a mortality rate of 114.8 per 100,000 people in 2013 [3]. There is a total of 2,633 strokes per 100,000 people in China, which is almost twice as many as that in the United States, and in 2019 the mortality rate of stroke rises to 149.49 per 100,000 people [4]. The most common type of stroke is acute ischemia stroke (AIS), which accounts for 80% of all cases [5]. Thrombolytic therapy is an active AIS treatment, but its clinical application is limited by such factors as strict contraindications, narrow treatment time windows, and critical side effects [6]. Therefore, there is an urgent need for much safer and more effective drugs with a wider therapeutic window to treat AIS.

cGMP-specific 3',5'-cyclic phosphodiesterase (PDE5) is a member of the PDE family, which is widely distributed in the nervous system [7, 8]. PDE5 can hydrolyze cyclic guanosine 3',5'-monophosphate (cGMP) and inactivate it. cGMP is involved in many physiological processes as a second messenger, especially in the production of neurons [9]. The classic PDE5 inhibitor sildenafil has been used to treat erectile dysfunction (ED) and AIS [10, 11] in clinic, but its serious side effects such as headache and increased risk of melanoma, have limited its clinical use [12]. Icariside II (ICS II) has recently been identified as a PDE5 inhibitor. Interestingly, ICS II has similar pharmacological effects to sildenafil in that it not only treats AIS but also inhibits melanoma. Therefore, ICS II seems to be a natural alternative or complement to sildenafil [13]. ICS II has shown a protective effect in preclinical studies of AIS. However, the mechanism of ICS II in the treatment of AIS is uncertain. The objective of this systematic review is to clarify the possible mechanisms of action of ICS II in the treatment of AIS.

ICS II (Chinese name is Baohuoside I) is the major pharmacological metabolite of Icariin, the main component of Epimedii [14]. And ICS II can be obtained from Epimedii under enzymatic hydrolysis conditions [14]. Herba Epimedii (commonly named as Yin-yang-huo in China) is a traditional herb or health food that is widely used in China for the prevention and treatment of various diseases. In vivo and in vitro studies have been conducted over the past few decades and found that ICS II can treat a variety of cancers including acute myeloid leukemia [15], prostate cancer [16], cervical cancer [17], etc. It has also been used to treat myocardial ischemic and reperfusion injury [18, 19], ED [20, 21] and many neurological disorders such as Alzheimer's disease [22–24], vascular dementia [25], and subarachnoid hemorrhage [26]. Especially in recent studies, ICS II has performed well in the treatment of AIS in the preclinical studies [27–30]. However, the underlying molecular mechanism of ICS II for the treatment of AIS is unclear.

The purpose of this preclinical study was to identify possible mechanisms of action of ICS II in AIS, to promote clinical translation of ICS II, as a dietary supplement to treat AIS or as an additional drug to enhance the effects of existing therapy. Furthermore, the potential pharmacological mechanism of ICS II for the treatment of AIS was explored using a network pharmacological approach. Molecular docking and molecular dynamics (MD) stimulation were further conducted to determine the binding efficiency of ICS II between important target genes.

## Material and methods

### Systematic review

#### Protocol and registration

We registered the protocol of this systematic review on the International Prospective Register of Systematic Review (PROSPERO). The registration number is CRD42022306291. The reporting of systematic reviews is based on the Preferred Reporting Items for Systematic Reviews and Meta-analyses (PRISMA). The entire PRISMA checklist can be found in Supplementary file 1.

#### Search strategy of systematic review

We have searched Chinese National Knowledge Infrastructure (CNKI), Chinese Science and Technology Periodicals Database (VIP), Wanfang, PubMed, Embase, and Web of Science databases. The published literature was searched with predetermined keywords. In general, keywords consisting of “baohuoside I”, “icarisid II”, “icariside II”, “ICS II”, “acute ischemic stroke” “Ischaemic Stroke”, “Stroke”, “cerebral ischemic stroke”, and “Cryptogenic Ischemic Stroke” were used, and adapted to each search engine. The search strategy was following Medical Subject Headings (MeSH) or keywords specific to each database: (1) Ischaemic Stroke; (2) icariside II; (3) Animal. An example of PubMed's keyword search strings was described in Supplementary file 2. The search strategy language has no restrictions. The literature was searched from January 6, 2022 to March 8, 2022, with no restrictions on date of publication. And the search strategy of identifying preclinical interventions in animal studies was used to explore the therapeutic effects of selective ICS II on AIS. The grey literature and extra sources of information were obtained from Opengrey database (www.opengrey.eu/).

#### Inclusion and exclusion criteria

##### Inclusion criteria

(1) Animal or population: Animal model of AIS, both genders, no age restriction. (2) Intervention: ICS II was administered as a treatment regardless of timing, regime, or administrative route. (3) Comparison: Studies with positive control and negative controls (with or without normal controls). (4) Outcome: neuronal protection, cerebral vascular protection, cerebral blood flow, memory performance, stroke lesion volume, oxidative stress, apoptosis, inflammation, etc. within brain tissue. (5) Other criteria: Reported original data, but there are no national and language restrictions. (6) Study design: No design restriction.

##### Exclusion criteria

(1) Animal/population: Animal model did not involve AIS. (2) Intervention: Adjuvant therapy was performed without an independent assessment of the effects of ICS II supplementation. (3) Other criteria: Reports of non-original data (i.e., not reviews, letters and abstracts, editorial, study protocol, conference abstracts, opinion piece).

#### Data extraction

The main literature search was conducted by a reviewer (ZiQiao Yu), and Yu also exported the title, abstract, keywords, author, publication year, and other information of the identified article to Microsoft Office Excel. In addition, two investigators (Xu Wang and Lifang Liu) have independently read all titles and abstracts to determine the eligibility of these in vivo studies. The full text of studies that may qualify was obtained, while two investigators (Xu Wang and Lifang Liu) screened each article using inclusion and exclusion criteria. Disagreements were resolved by another investigator (Jinhua Li). All reference lists of included articles were screened by a researcher (Xu Wang) to obtain possible additional eligible studies. Due to the diversity of methods, there was no effort to pool quantitative data from different studies. The reviewer (Xu Wang) explicitly extracts all the information from each retrieved report, the details of the article, sex, animal model, induction model dosage, frequency, outcome measurements added to a predefined excel table for data management. Two reviewers (Lifang Liu and Jinhua Li) re-examine the extracted information to maintain consistency and relevance of the data. For incomplete and missing data, the author contacted in person to request further specific information. The outcomes are the same as the outcome in inclusion criteria in the previous section.

#### Risk of bias and quality assessment

The risk of bias for all reports was rigorously evaluated independently by two reviewers (Xu Wang and Jinjian Li), using the SYRCLE Risk of Bias (RoB) tool. This tool was used to assess the methodological quality of preclinical studies, with 10 entries associated with 6 biases. The reviewers assesse the risk of bias in each report by coding unclear, no, and yes for the risk of bias, respectively, indicating insufficient detail, high risk, and low risk. Finally, two reviewers resolve any conflict on the same issue through discussion until a consensus is reached or a third auditor (Jinhua Li) is consulted. Any study with three sources of high risk of bias by SYRCLE ROB was excluded.

Quality assessment was used checklist of the Animal Research: Reporting in Vivo Experiments (ARRIVE) guidelines, a tool to evaluate the risk of experimental bias, including the use of indicators such as randomization and blinding to assess the bias of animal selection and outcome evaluation [31] and the Animal Data for Experimental Studies meta-analysis and retrospective collaboration (CAMARADES) [32].

### Network pharmacological analysis

#### Collection of targets for ICS II and AIS

The AIS targets were retrieved from GeneCards (www.genecards.org/) and GEO databases (www.ncbi.nlm.nih.gov/geo/). The AIS-related microarray datasets GSE16561 was downloaded from the GEO database. Following are the steps taken to process the data using R project. First, probes are mapped to genes and no-load probes are removed. The expression level of a gene was determined by a random value within the duplicate gene if more than one probe was mapped to that gene. After normalizing gene expression to the Log2 transformed quantile-normalized signal intensity, further analysis could be performed. The differentially expressed genes (DEGs) were identified using LIMMA package with log2 fold change (LogFC) > 0.5 or LogFC <  − 0.5 and adjusted *P* < 0.05. The remove batch effect function in the LIMMA software package was used to eliminate the effect of sex on gene expression.

**Fig. 1:**
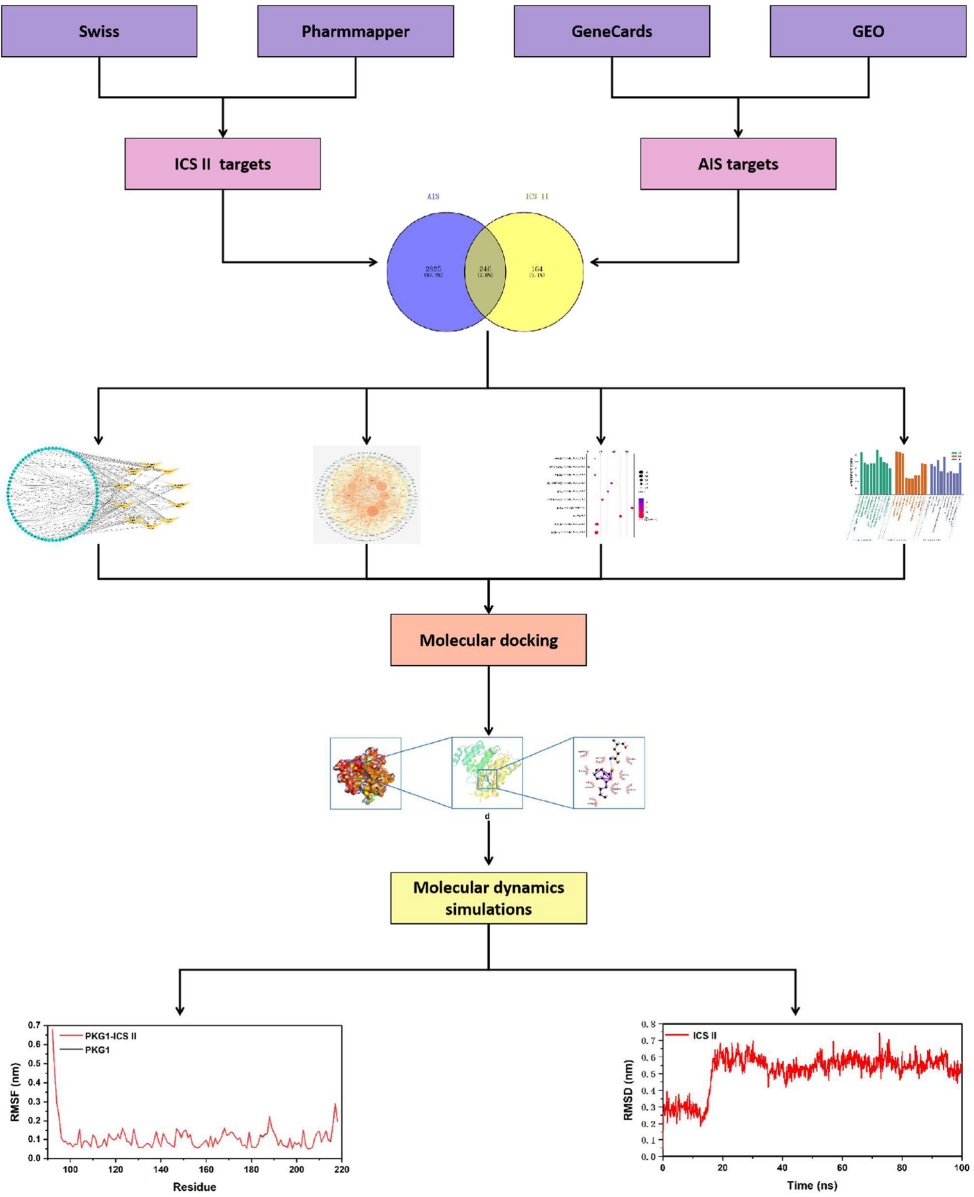
The workflow of this study

The ICS II-associated targets were predicted using Pharmmapper (www.lilab-ecust.cn/pharmmapper/) and the SwissADME database (www.swissadme.ch/). In addition, these targets were also obtained from the proteins which have been validated to be differentially expressed by western blot from the literature included in the systematic review. The flowchart for network pharmacology in this study is shown in Fig. 1.

#### Protein–protein interaction (PPI) network construction and analysis

String 11.0 (string-db.org/) is a database which is used to predict protein interactions. The ICS II-AIS targets were imported into String for PPI analysis, and a PPI network with the species is set to "Homo" and a confidence level of > 0.9 is set as a condition. Finally, the PPI network was built by Cytoscape 3.7.2 (Cytoscape.org/).

#### GO and KEGG enrichment analysis

The target genes were imported into the online website Metascape (metascape.org/) for KEGG and GO enrichment analysis. The purpose of the GO and KEGG enrichment analysis was to analyze the potential biological pathways and functions associated with the ICS II-AIS targets, and *P* < 0.05 is the limit of the results.

#### Construction of the target-pathway network

The target-pathway network was created by Cytoscape 3.7.2 software. In the network, edges and nodes are both two critical elements of a network, “node” represents the pathways and targets in ICS II, and “edge” indicates the connection between the pathways and targets [33]. The "degree" is a parameter used to assess the importance of potential targets in network pharmacology.

#### Molecular docking verification

The 2D structure of ICS II and the targets were gotten from the PubChem (pubchem.ncbi.nlm.nih.gov/) database and RCSB protein (www.pdb.org/) database. Ligand binding models smaller than 3 Å are preferred, and the 2D structure is imported into Pymol (pymol.org/2/) software for ligand dehydration and hydrogenation, and then imported into AutoDockTools to set the parameters of the docking grid box of all targets. We used Autodock Vina software for molecular docking, selected the molecular docking conformation with the lowest binding energy, and observed the binding effect through pymol software.

#### Molecular dynamics simulations

The initial structure of PRKG1 (PDB ID: 3OCP) was taken from PDB (www.rcsb.org/) database. Substrate was docked into the active site of protein using AutoDock Vina tool [34] in Chimera [35]. In the MD simulations that followed, the docked pose with the highest docking score was used. The substrate was obtained using the general AMBER force field (GAFF) [36], while part of the atomic charge was obtained by the RESP method of Multitwon [37]. The parmchk utility from AMBERTools was used to generate the missing parameters for the ligands. Na^+^ and Cl^−^ ions were added into the systems to neutralize the total charges of the systems. Finally, we dissolved the resulting system in a rectangular box containing TIP3P [38] waters, limiting the extension from the protein boundary to the minimum cutoff point of 15 Å. All of the MD simulations were employed for the protein by the Amber ff14SB force field [39]. The initial structure was minimized by the combined steepest descent and conjugate gradient method. As a next step, the systems were gently annealed from 10 to 300 K under canonical ensembles for 0.5 ns with a weak constraint of 10 kcal/mol/Å. An isothermal-isobaric ensemble with a target temperature of 300 K was used to equilibrate density in 0.5 ns and we used Langevin-thermostat and Berendsen barostat with collision frequencies 0.002 ns and pressure relaxation times 0.001 ns to achieve the target pressure of 1.0 atm. When all complex systems were properly minimized and equilibrated, all MD runs were completed within 100 ns. After proper minimization and equilibration, an effective MD simulation runtime of 100 ns was used for all complex systems. The MD simulations were performed using Gromacs (2019.6) [40].

## Results

### Systematic review

#### Study characteristics

A total of 308 publications were initially identified for this study. A number of 283 papers were excluded which were filtered by titles and abstract, and 25 papers were excluded after reading through the full text. There are no new publications in the reference list of the selected articles. The 12 studies that were considered eligible for systematic review took disparate methodology. Therefore, statistical pooling is impractical. This heterogeneity is attributed to the study characteristics of different AIS models and treatment types (different doses of ICS II), different routes of administration, and duration of treatment. Although no date limit is set during the literature search process, the final report included in the systematic review was published between 2014 and 2021. Of the 12 included studies, three (25%) were published in 2020, two were published in 2014 (16.7%) and 2019 (25%), and one each was published in 2016, 2017, 2018 and 2021.

#### Experimental animals

**Fig. 2:**
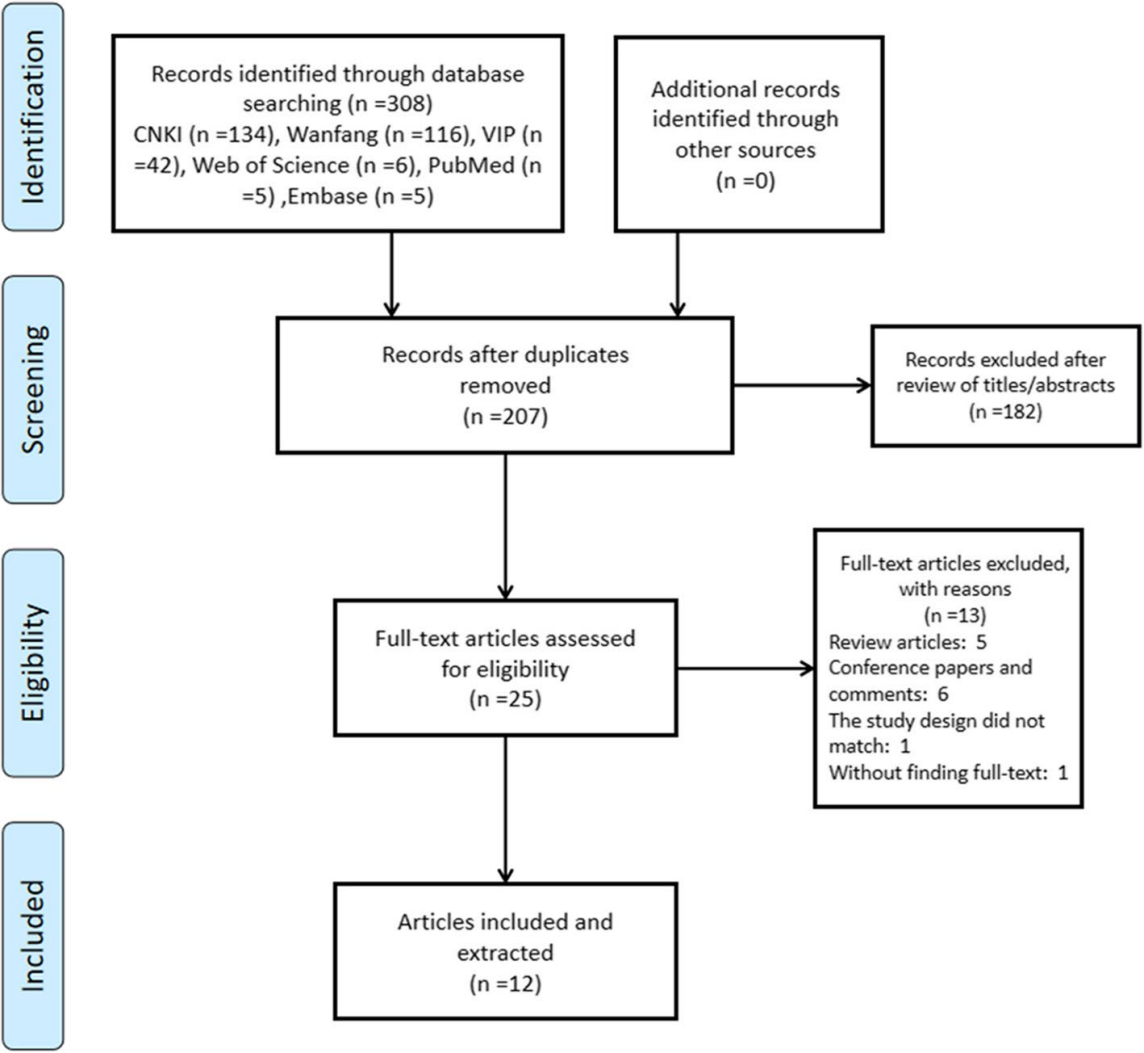
The flow diagram of search, screening and study selection of the systematic review

The remaining 12 studies included in this systematic review evaluated the results of 1993 animals (1609 rats, 300 mice, and 84 gerbils). The flowchart for this systematic review is shown in Fig. 2. Details about the animal models and experiments are shown in Table 1.

**Table 1:** Overview of included studies and results of ICS II in an animal model of acute ischemic stroke

Study (years) Language	Species	Model and duration	Treatment	Effects	Functional outcomes
Gao et al. (2019) English	Sprague–Dawley rats, male	MCAO, 2 h	4, 8, and 16 mg/kg, twice a day from Day 1 to Day 7	↑Memory; ↓Autophagy	Stroke volume; Brain water content
Deng et al. (2016) English	Sprague–Dawley rats, male	MCAO, 2 h	10 and 30 mg/kg, twice a day for 3 consecutive days pretreatment	↓Inflammation; ↓Apoptosis	Stroke volume
Liu et al. (2020) English	Sprague–Dawley rats, male	MCAO, 2 h	16 mg/kg twice a day for 3 days	↓Apoptosis; ↓BBB; ↓Neuronal injury	Stroke volume
Yan et al. (2014) English	Mongolian gerbils, male	MCAO, 30 min	20 mg/kg at 2 h before ischemia, and 6, 24, 48, 70 h after the beginning of reperfusion	↓Apoptosis; ↓Oxidative stress; ↓Neuronal injury	Cerebral bloodflow
Ma et al. (2021) Chinese	C57BL/6 mice, male	MCAO, 2 h	5, 10, and 20 mg/kg twice a day for 7 days pretreatment	↓Oxidative stress	Stroke volume; Cerebral bloodflow
Deng et al. (2019) Chinese	Sprague–Dawley rats, male	BCCAO	4, 8, and 16 mg/kg for 28 days	↑Memory	
Long et al. (2018) Chinese	Sprague–Dawley rats, male	MCAO, 2 h	4, 8,and 16 mg/kg twice a day for 3 consecutive days	↓Oxidative stress; ↓Autophagy	Stroke volume; Brain water content
Long et al. (2017) Chinese	Sprague–Dawley rats, male	MCAO, 2 h	4, 8, and 16 mg/kg for 3 days	↓Oxidative stress	Stroke volume; Brain water content
Li et al. (2019) English	Sprague–Dawley rats, male	MCAO, 2 h	5, 10, and 20 mg/kg for 1 day	↓Oxidative stress	Stroke volume; Brain water content
Gao et al. (2020) Chinese	Wistar rats, male	MCAO, 2 h	4, 8, and 16 mg/kg twice a day for 3 consecutive days	↓Inflammation; ↓Oxidative stress; ↓BBB; ↓Neuronal injury	Stroke volume; Brain water content
Xiong et al. (2014) Chinese	Sprague–Dawley rats, male	MCAO, 2 h	10 and 30 mg/kg twice a day for 3 days pretreatment		Stroke volume
Liu et al. (2020) Chinese	Sprague–Dawley rats, male	MCAO, 2 h	16 mg/kg for 3 days	↓Apoptosis; ↓BBB	Stroke volume; Cerebral bloodflow

The animals were used differently in these included studies. Ten of the studies used rats (84%), and the other two studies selected Mongolian gerbils (8%) and C57BL/6 mice (8%). All studies selected animals of male sex to avoid the influence of sex on the experiment, and some studies have shown a therapeutic effect of estrogen in ischemic stroke [41]. The vast majority of studies (92%) selected the middle cerebral artery occlusion (MCAO) model, and only one study (8%) selected the bilateral common carotid artery occlusion (BCCAO) model.

#### Intervention characteristics

For the single administration of ICS II, the maximum dose was 40 mg/kg (average dose 25.2 mg/kg) and the minimum dose was 4 mg/kg (average dose 7.3 mg/kg). At the maximum dose administration, no toxic injury was found in the animals, which indicated that ICS II has a good safety profile. The general duration of treatment was 1–7 days after induction of AIS, with 8 studies (67%) administering bites for 3 days, two studies (17%) administering for seven days, one study administering for 1 day (8%) and the other for 28 days (8%). In summary, the doses, frequencies, and timing of administration were essentially different in the included studies.

#### Outcome

We make decisions and conclusions about the results (reported results, limitations, etc.) based on trends in most studies. The studies included in the review used a wealth of outcome measures. The main objective of the pre-clinical studies was to evaluate the neuroprotective effect of ICS II in various ischemic models. In general, these studies using ICS II showed positive outcomes in the form of reduced cerebral infarct size and reduced neurological deficits after treatment. One study [30] did not describe infarct size, but the rest of the studies (*n* = 11, 92%) showed that ICS II reduced infarct size and neurological deficits in AIS, and negatively correlated with drug dose range. Three studies (25%) concluded that cerebral blood flow was improved. The conclusions of two studies suggest reduced permeability of blood–brain barrier (BBB). Five studies (42%) have shown that ICS II reduces brain water content. Two studies (16%) have shown that ICS II weakens the inflammatory response. The results of six studies (50%) were antioxidant stress of ICS II. Six studies (50%) concluded that cell death was reduced through anti-apoptotic and anti-autophagy. Another study (8%) found that ICS can improve memory.

#### Risk of bias and quality assessment

Risk of bias assessment was performed using the SYRCLE RoB tool (Table 2). In all domains, no studies were assessed as a high risk of bias. Eleven studies have shown that animal groupings are randomized, although some studies do not give specific details about how to proceed with randomization. All studies did not have biased effects on baseline features. Only one study (8%) explicitly reported randomly raising animals during the experiment. Two studies (16%) reported on the blinding of evaluators in the intervention and control groups. Six studies (50%) reported randomly selected animals for outcome evaluation, and five studies (43%) reported that the evaluators were blind, and the loss bias in all studies was unclear. None of the studies had additional biases.

**Table 2:** SYRCLE Risk of Bias Assessment for included studies

Reference	Random Sequence Generation	Groups Similar at Baseline	Allocation Concealment	Animals Random Housing	Blinding of CAREGIVERS and/or Examiners	Random Outcome Assessment	Blinding of Outcome Assessor	Incomplete Outcome Data Addressed	Free from Selective Outcome Reporting	Free from Other Bias?
Gao et al	**Y**	**Y**	**Y**	**Y**	**U**	**U**	**U**	**Y**	**Y**	**Y**
Deng et al	**U**	**Y**	**Y**	**U**	**U**	**Y**	**Y**	**Y**	**Y**	**Y**
Liu et al	**U**	**Y**	**U**	**U**	**U**	**U**	**U**	**Y**	**Y**	**Y**
Yan et al	**U**	**Y**	**Y**	**U**	**U**	**U**	**U**	**Y**	**Y**	**Y**
Ma et al	**U**	**Y**	**Y**	**U**	**U**	**U**	**U**	**Y**	**Y**	**Y**
Deng et al	**U**	**Y**	**Y**	**U**	**U**	**U**	**U**	**Y**	**Y**	**Y**
Long et al	**U**	**Y**	**Y**	**U**	**Y**	**Y**	**Y**	**Y**	**Y**	**Y**
Long et al	**U**	**Y**	**Y**	**U**	**U**	**U**	**U**	**Y**	**Y**	**Y**
Li et al	**U**	**Y**	**Y**	**U**	**U**	**U**	**U**	**U**	**Y**	**Y**
Gao et al	**U**	**Y**	**Y**	**U**	**U**	**Y**	**Y**	**Y**	**Y**	**Y**
Xiong et al	**U**	**Y**	**Y**	**U**	**U**	**Y**	**Y**	**Y**	**Y**	**Y**
Liu et al	**U**	**Y**	**Y**	**U**	**U**	**Y**	**Y**	**Y**	**Y**	**Y**

Quality assessment of pre-clinical studies showed that 11 papers reported random grouping, but did not specify the method of grouping, and there were no differences in body weight between groups of animals. The average CAMARADES score is 5 out of 10, with a median score of 5. The median score of ARRIVE criteria scored 21 out of 38, and the average score is 20. Specific scoring information can be found in Supplementary file 3.

### Network pharmacological analysis

#### Targets of ICS II and AIS

**Fig. 3:**
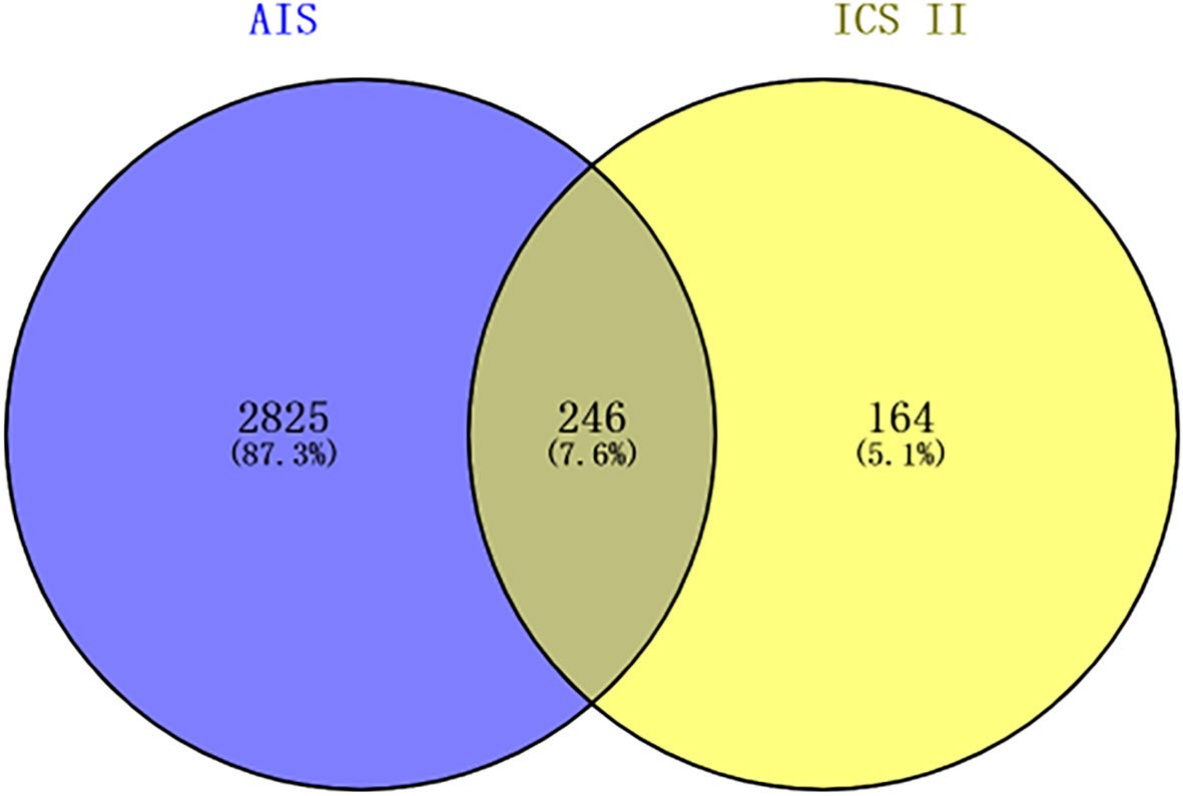
Intersection targets of ICS II and AIS

Three thousand seventy-one AIS targets were obtained from Genecards and GSE16561. We integrated the acquired targets and removed duplicate targets, leaving 409 targets. Then, the name of each target protein ID is queried through the UniProt (uniprot.org/) database, and the species is selected as "Homo" to obtain each target’s gene ID. The resulting data were then the targets of ICS II. Finally, we used VENNY2.1 (www.bioinfogp.cnb.csic.es/tools/venny/) to obtain 246 target genes for both ICS II and AIS, as shown in Fig. 3. The details of targets can be obtained from Supplementary file 4.

#### ICS II-AIS PPI Network

**Fig. 4:**
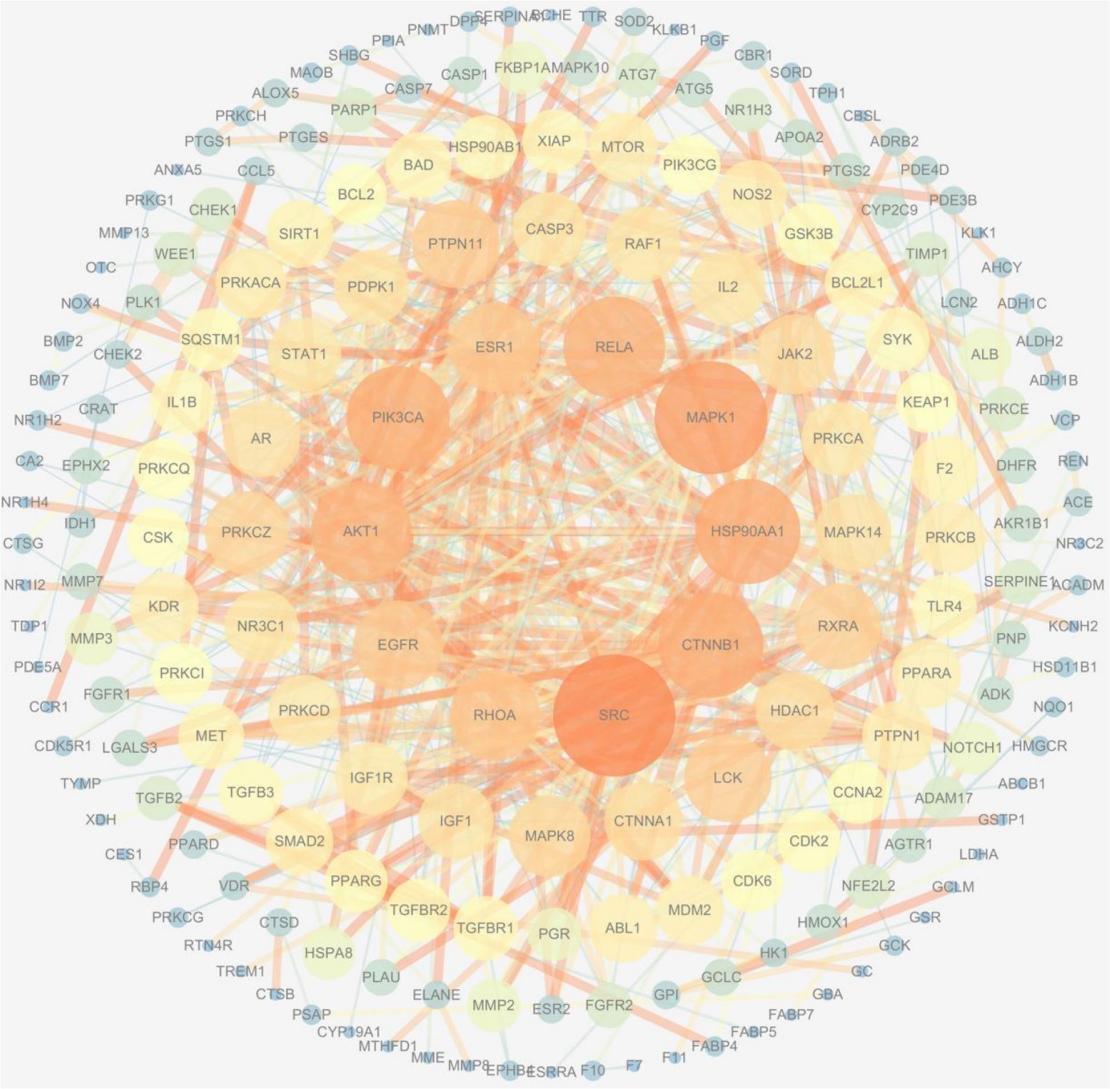
The network diagram of core targets and non-core targets. The nodes, closing to the center and in dark color represent that they play a key role in the whole network

The PPI network diagram was constructed using the “generate styles from statistic in the tools” in Cytoscape (Fig. 4). The PPI network contained 199 nodes (47 nodes were excluded on the condition of confidence > 0.9) and 686 edges. The higher the degree value, the more important the node is in the network [42]. The closer the nodes from the outside to the inside, the more important the target is represented. The results showed that SRC, CTNNB1, HSP90AA1, MAPK1, RELA, ESR1, PI3KCA, AKT1, EGFR, and RHOA had higher degree. This indicated that these targets may play an important role in the treatment of AIS by ICS II. The information of the top 10 targets of ICS II is shown in Table 3.

**Table 3:** The specific information of the top 10 core targets from topological analysis of 246 targets

Gene symbol	Protein name	Degree (DC)
SRC	Proto-oncogene tyrosine-protein kinase Src	46
CTNNB1	Catenin beta-1	40
HSP90AA1	Heat shock protein HSP 90-alpha	35
MAPK1	Mitogen-activated protein kinase 1	34
RELA	Transcription factor p65	34
ESR1	Estrogen receptor, ER	33
PI3KCA	Phosphatidylinositol-4,5-bisphosphate 3-kinase	32
AKT1	RAC-alpha serine/threonine-protein kinase	26
EGFR	Epidermal growth factor receptor	25
RHOA	Transforming protein RhoA	25

#### GO and KEGG enrichment analysis

**Fig. 5:**
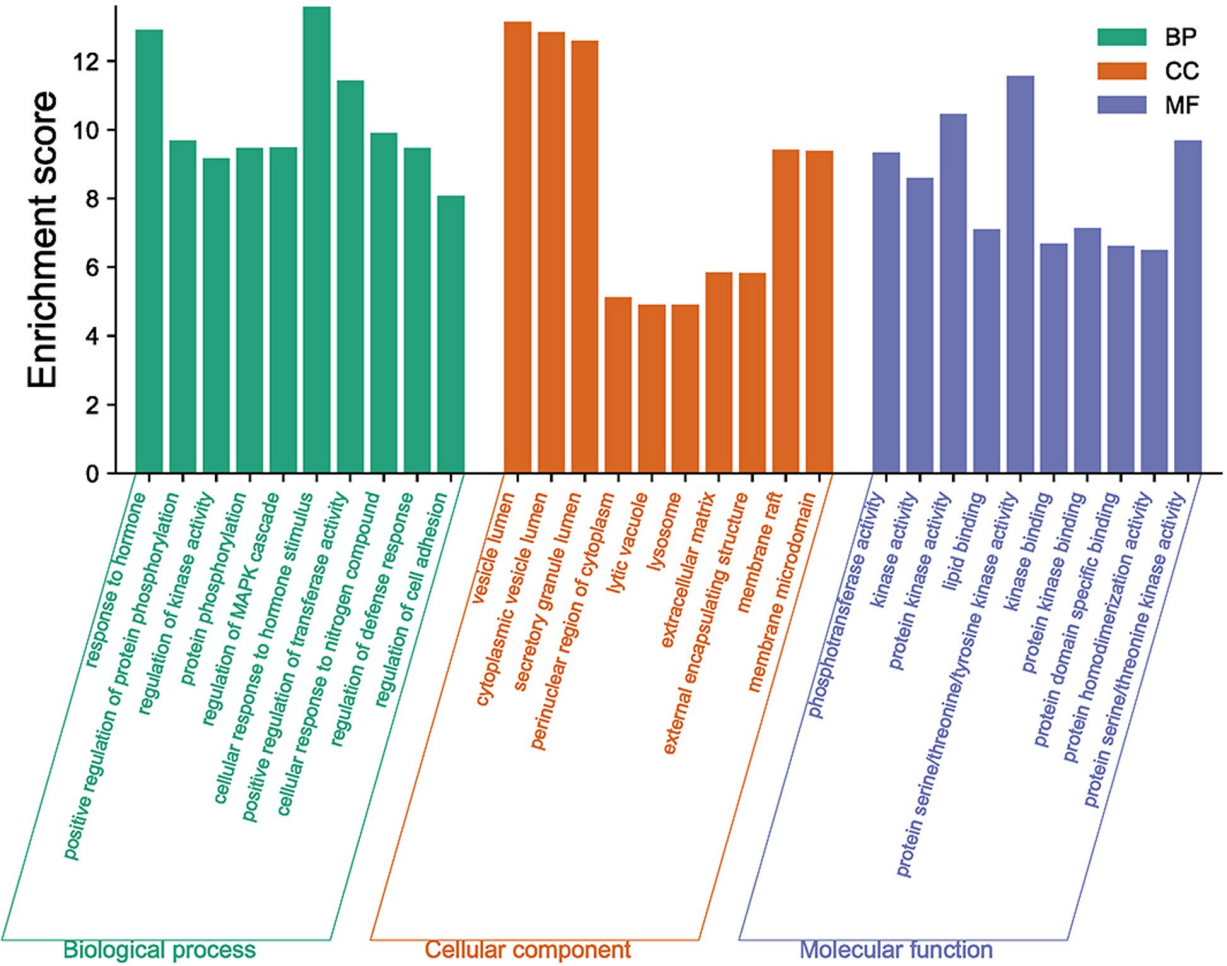
GO enrichment analysis of ICS II in treating AIS. GO items and Enrichment score are represented by the x-axis and y-axis, respectively

GO analysis of the 246 relevant targets is specifically divided into three parts, including biological processes (MF), cell composition (CC), and molecular function (MF), which is shown in Fig. 5. The top 10 GO terms were phosphotransferase activity, alcohol group as acceptor, kinase activity, protein kinase activity, lipid binding, protein serine/threonine/tyrosine kinase activity, kinase binding, protein kinase binding, protein domain specific binding, protein homodimerization activity, and protein serine/threonine kinase activity. For BP ontology, the top ten GO terms were response to hormone, positive regulation of protein phosphorylation, regulation of kinase activity, protein phosphorylation, regulation of MAPK cascade, cellular response to hormone stimulus, positive regulation of transferase activity, cellular response to nitrogen compound, regulation of defense response, and regulation of cell adhesion. For CC, the top ten were vesicle lumen, cytoplasmic vesicle lumen, secretory granule lumen, perinuclear region of cytoplasm, lytic vacuole, lysosome, extracellular matrix, external encapsulating structure, membrane raft, and membrane microdomain.

**Fig. 6:**
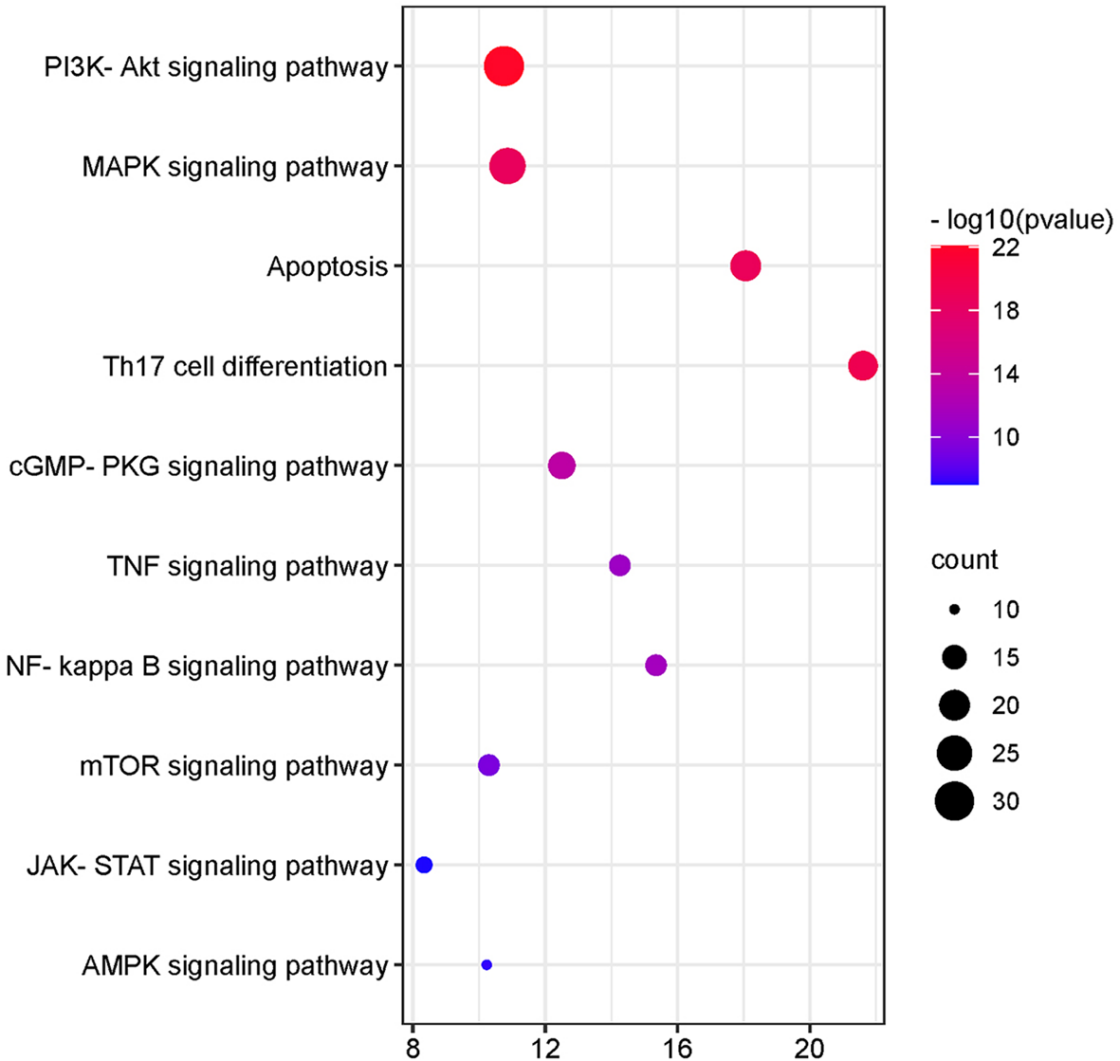
Top 10 enrichments of KEGG analysis with ICS II in treating AIS

Based on an understanding of the pathogenesis of AIS, we have a complete AIS pathway. The top 10 KEGG (We removed pathways not associated with AIS, such as pathways in cancer, Hepatitis B, Toxoplasmosis, etc.) signaling pathways of ICS II is shown in Fig. 6. The top 10 KEGG pathways were Th17 cell differentiation, Apoptosis, MAPK, cGMP-PKG, NF-kappa B, TNF, mTOR, AMPK, PI3K-Akt, and JAK-STAT signaling pathway.

#### Target-pathway Network

**Fig. 7:**
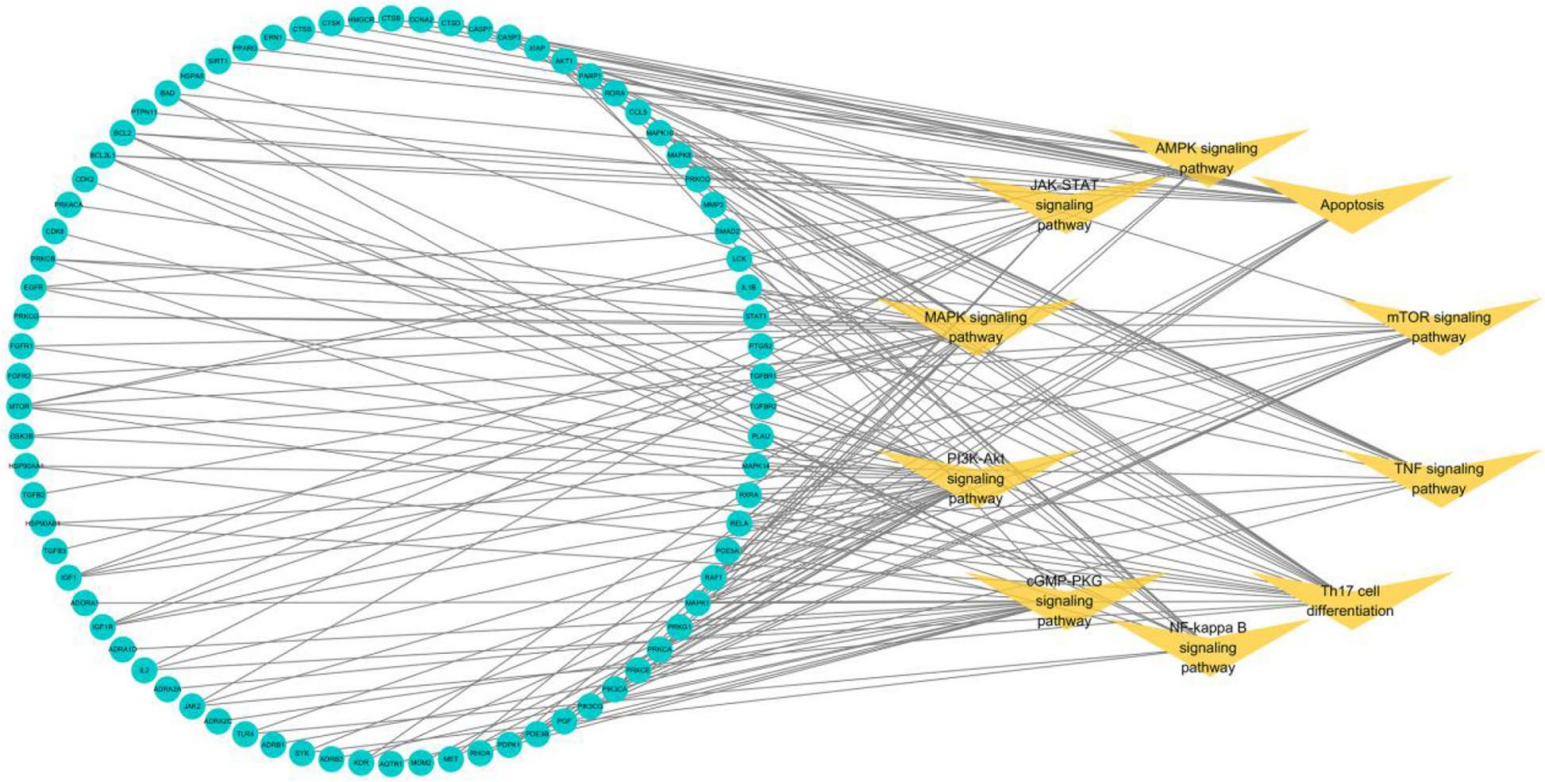
Targets-pathway network of ICS II in treating AIS. The green nodes represent the target genes and yellow nodes represent related pathways

**Fig. 8:**
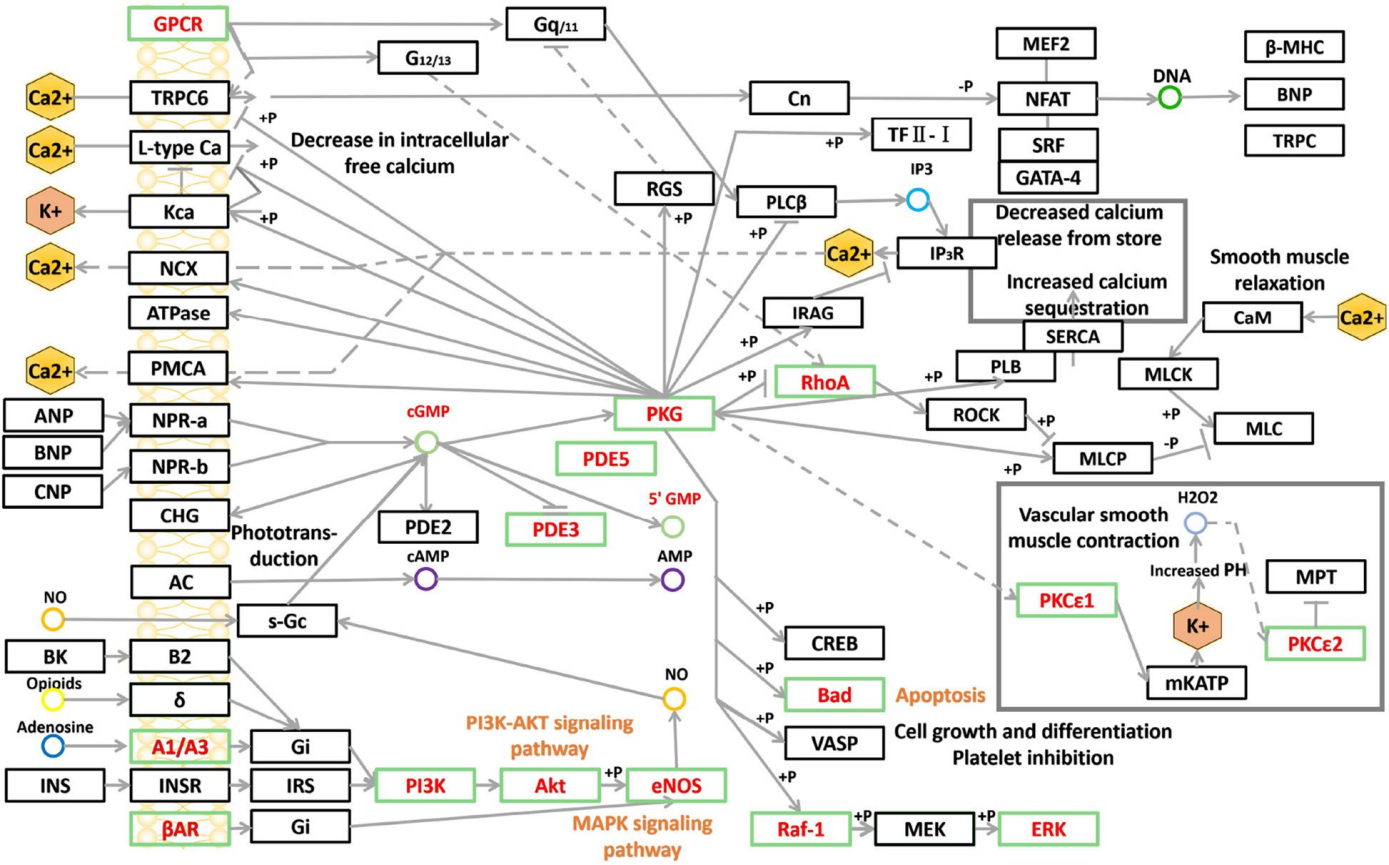
Pathway map of ICS II against AIS. The key targets of ICS II in the treatment of AIS were shown as rose red in the cGMP-PKG signal pathway

Further, a target-pathway was established to reveal the connections between ICS II targets and top 10 pathways, as shown in Fig. 7. One target map to multiple pathways, and multiple targets mediate one pathway simultaneously, implying that these targets may regulate the interactions and crosstalk of different pathways. We found that the cGMP-PKG signaling pathway is a very important mechanism for ICS II in treatment of AIS (Fig. 8). Interestingly, enrichment analysis showed crosstalk and cascade between autophagy and the cGMP-PKG, PI3K-Akt, and MAPK signaling pathway.

#### Predicted binding of ICS II to significant target proteins

**Fig. 9:**
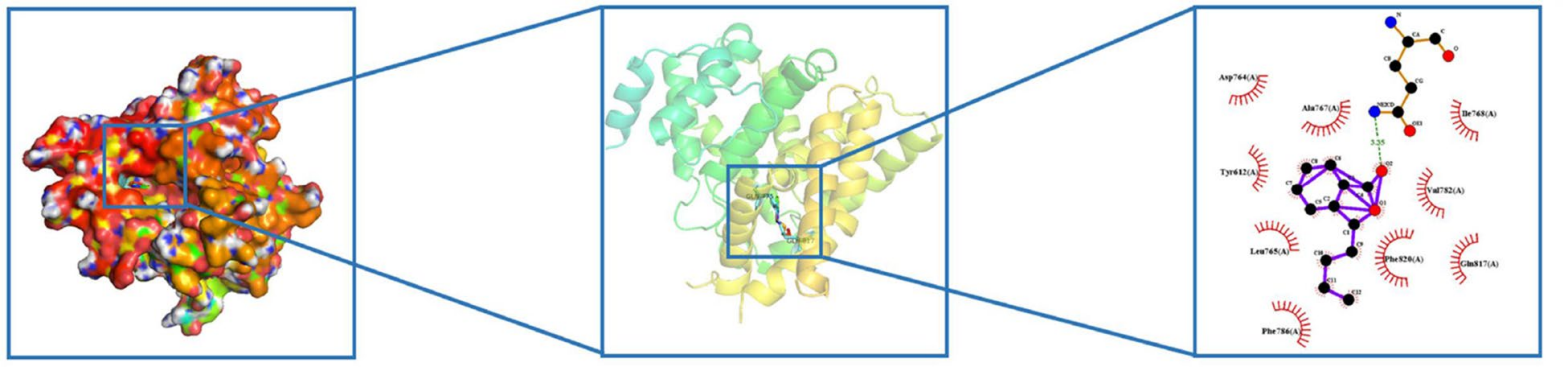
Molecular docking diagram of PRKG1 and ICS II

The smaller the molecular docking result, the stronger the binding between the receptor and the ligand, and all the molecular docking results are less than -5.0 kJ/Mol, indicating that ICS II has a strong binding effect with key proteins such as PKG1 (Fig. 9). All docking results were shown in Table 4.

**Table 4:** Target docking parameters and corresponding calculation results

Component	Target (gene symbol/protein name)	Binding energy (kJ/Mol)
ICS II	AKT1/RAC-alpha serine/threonine-protein kinase	-6.9
ICS II	BAD/Bcl2-associated agonist of cell death	-7.4
ICS II	MAPK1/Mitogen-activated protein kinase 1	-8.9
ICS II	PIK3CG/Phosphatidylinositol 4,5-bisphosphate 3-kinase catalytic subunit gamma isoform	-9.6
ICS II	PRKG1/cGMP-dependent protein kinase 1	-9

#### Molecular dynamics simulations

**Fig. 10:**
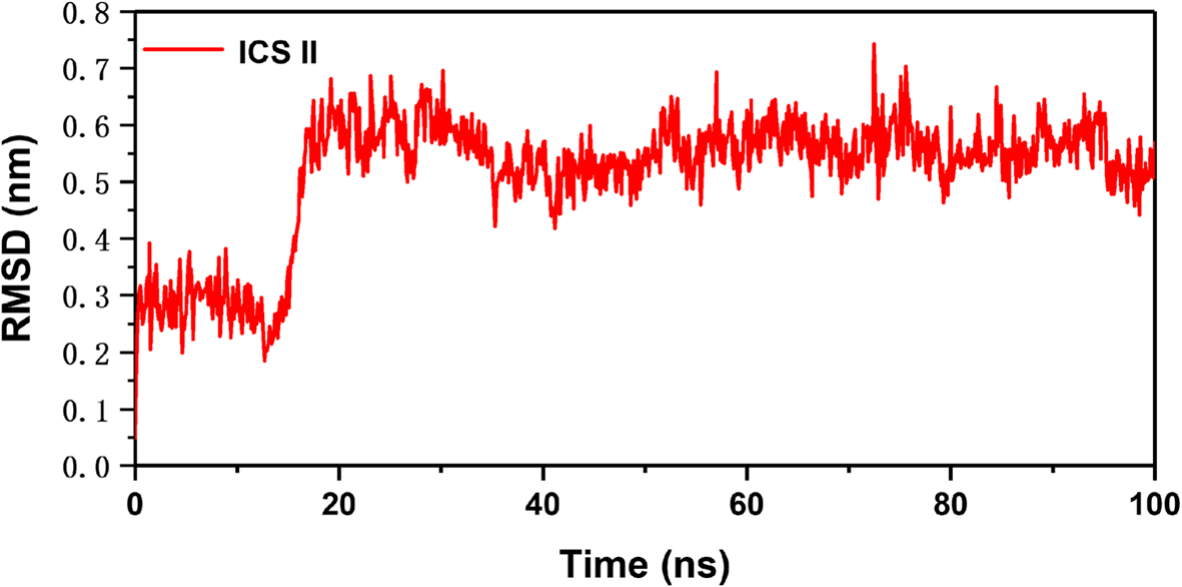
RMSD of ICS II. The conformation of all systems has reached a steady-state because the RMSD value fluctuates for the original structure of complex within 0.2 nm which indicates the stability of the structures

**Fig. 11:**
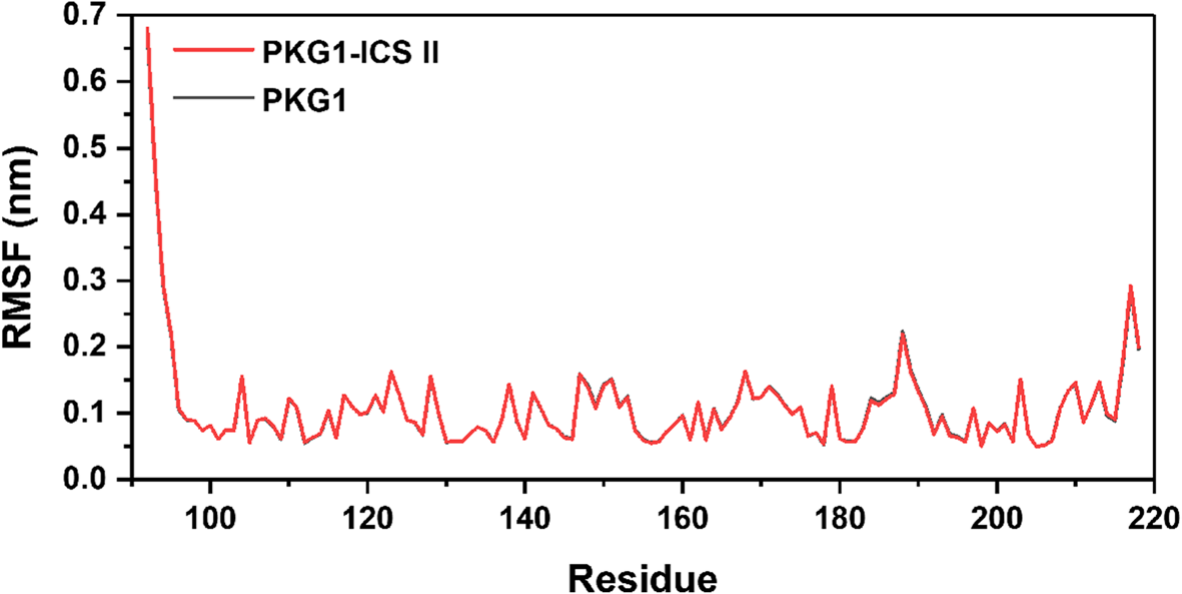
RMSF of ICS II. Most of the residues had low fluctuation values in other regions, which indicated that the residues are stable in binding to the protein

To further study the interaction between ICS II and PKG1, we performed a 100 ns MD simulation. In addition, the RMSD from the average structure of backbone atoms for each MD trajectory was calculated as well for exploring the "position stability" of the original conformation for each complex. Figure 10 was plotted the RMSD of backbone atoms of the complex system and the result showed that after 18 ns, the conformation of all systems has reached a steady-state because the RMSD for the original structure of complex reached at about 5 Å, which indicates the stability of the structures. As shown in Fig. 11, root mean square fluctuation (RMSF) has provided details about the structural flexibility of individual residues in a protein.

## Discussion

In this study, we first conducted a preclinical systematic review of ICS II for the treatment of AIS to assess the role of ICS II as a potential therapeutic target for stroke. The conclusion is based on the trend of most studies. While all of the studies retrieved were released after 2014, there was no application date limit in any database, which confirms that the efficacy of ICS II in AIS has been extensively studied over the past decade. Most studies in this area have been published since 2019, which explains the rapid growth in interest in this area.

The vast majority of studies used the MCAO model, which reperfusion followed two hours of ischemia. And all the animals in the experiments were males. Although this avoids the confounding factors that estrogen comes with, this also leads to unclear roles of ICS II in female AIS animals, which needs to be confirmed in future experiments.

ICS II has showed good neuroprotective effects in AIS animals and did not show toxicity at a high dose of 40 mg/kg, indicating that ICS II has a high safety profile. And the side effects of long-term use of ICS II and adverse reactions with other drugs are not clear. This means that there is still a lot of work to run for the application of ICS II in the clinic.

The results of the systematic review of preclinical studies showed that ICS II reduced cerebral infarct size, neuronal and blood–brain barrier damage through anti-inflammatory, anti-apoptotic and anti-oxidative stress in the treatment of AIS. Deng’s study showed ICS II treatment has improved memory after AIS [27], indicating that memory impairment after AIS can be reversed by ICS II. Some studies [29, 30] showed that neuronal damage after ischemia and hypoxia could be attenuated by ICS II. ICS II treatment reduced cell loss in the hippocampus, striatum and cortex, and the reduction in neuronal damage may be due to the fact that ICS II treatment reduced the microcirculatory dysfunction caused by AIS as well as the reduction in red blood cell velocity and cerebral blood flow. There were several potential mechanisms of action of ICS II on AIS in these studies. (1) BBB disruption is an important pathophysiological process in AIS, and reducing permeability of BBB can prevent the transformation of AIS condition to malignant brain edema and hemorrhagic [41]. Liu et al. [29] found that ICS II reduces permeability of BBB after AIS. This may be related to the down-regulation of Bax protein and cleaved-caspase3, and up-regulation of tight junction-related protein expression. The high dose of ICS II (16 mg/kg) was close to the BBB protection effect of nimodipine after AIS. (2) The internal environmental homeostasis of the brain is essential for the normal functioning of the central nervous system. Recent study by Gao J et al. [28] have shown that ICS II can reduce the water content of AIS brain. Increased brain water content disrupts this homeostasis and myelin integrity, exacerbates BBB permeability, and exacerbates brain tissue damage [43]. The decrease in brain water content is beneficial for neurological recovery after AIS. (3) ICS II may be regulated through PDE 4 and PDE 5 levels and the TGF-β1/Smad pathway. Interestingly, ICS II (8, 16 mg/kg) significantly decreased PDE 4 and PDE 5 concentrations, with no change at 4 mg/kg, suggesting that treatment of ICS II on AIS is clearly dose-dependent. ICS II could directly bind to PDE5 and GSK-3β and reduce brain injury after AIS by interfering with the PKG/GSK-3β autophagic axis and also by decreasing LC3B and Beclin1 protein expression. (4) ICS II may attenuate cell death in the process of AIS. Gao J et al. [28] found that ICS II inhibited neuronal cell autophagy after AIS. Several studies by Deng Y et al. [27, 29, 30] found that neuronal apoptosis after ischemia and hypoxia could be inhibited by ICS II, and ICS II significantly ameliorates post-AIS injury by regulating MMP9/TIMP1 homeostasis and increasing Bax and cleaved-caspase3 expression and further inhibiting neuronal apoptosis through caspase 3-dependent apoptotic pathway, with reduced Bcl-2/Bax expression. (5) Yan et al. [30] found that ICS II reduces oxidative stress after AIS. ICS II activated the Nrf2/HO-1 signaling pathway in brain tissue, reducing the levels of reactive oxygen species (ROS) and malondialdehyde (MDA) and increasing superoxide dismutase (SOD) activity to reduce oxidative stress. (6) Inflammation after neuronal injury could be attenuated by ICS II. ICS II inhibited the expression of IL-1β and TGF-β1 and also downregulated miR-141-3p levels to activate the Notch/Nrf2 axis to reduce inflammation in AIS.

The good efficacy and low side effects of ICS II make it very promising in the treatment of AIS [44]. However, for the included studies, the ARRIVE and CAMERADES methodological scores were generally low. And all included studies were not at low risk of bias by SYRCLE RoB tool which may have contributed to biased results.

We have further validated our findings with a network pharmacology and molecular docking approach. Among the results of KEGG, the cGMP-PKG signaling pathway attracted our attention. Although the cGMP-PKG signaling pathway is not ranked in the top, but many pathways have crosstalk and cascade relationship with the cGMP-PKG signaling pathway. Moreover, ICS II acts as an inhibitor of PDE5 to regulate cGMP levels, and its mechanism of action should be closely related to the cGMP-PKG signaling pathway. The cGMP-PKG signaling pathway plays an important role in many diseases including tumors [45, 46], myocardial infarction [47, 48] and neurological disorders. In recent studies, the cGMP-PKG signaling pathway has been shown to play a neuroprotective role after alcohol toxicity [49], Alzheimer's disease [50, 51], Parkinson [52], and acute ischemic stroke [53]. In neurological diseases, such as stroke and Alzheimer's disease, there are crosstalk and cascade responses between PKG and PI3K [35, 36], VEGF [34], MAPK [38], and apoptosis [37]. These findings suggested that inhibition or activation of the cGMP-PKG signaling pathway has an important regulatory role in neurological diseases [28, 54]. In particular, changes in PKG (gene symbol PRKG1) expression are more important for the above pathway to cause cascade responses, but more experimental evidence is needed. In particular, PKG has been shown to be an important target for protection against stroke, it can regulate CREB, GSK-3β and other proteins to reduce autophagy and apoptosis-induced neuronal damage after AIS [13, 28, 53].

However, the expression of PKG is influenced by the level of cGMP. During the progress of AIS, the local cerebral blood flow is reduced due to arterial or small artery occlusion. In the ischemic core, neurons are lost, but neuronal death can be saved if sufficient blood flow reaches the surrounding ischemic semidark zone in time. The cGMP-PKG signaling pathway has been shown to promote angiogenesis [55, 56] and increase vascular elasticity [57]. Changes in intracellular cGMP concentration translate into membrane potential, and increase intracellular Ca^2+^ and K^+^ concentrations, which cause smooth muscle contraction and regulate vasodilation [58–60]. These contribute to the restoration of blood supply to protect neurons in the ischemic semidark zone. PKG is essential for the relaxation of vascular smooth muscles. This enzyme can be activated by cGMP and oxidation and has many targets for the regulation of vascular tone [61]. Moreover, it inhibits platelet adhesion and aggregation by regulating NO, cGMP and PKG levels [62]. Higher levels of cGMP are found in shear-stressed platelets that limit thrombus formation around thrombi [63]. As well, the cGMP-PKG signaling pathway is beneficial to axons, neural networks, and synaptic plasticity [64, 65]. These mechanisms are very beneficial in limiting the deterioration and recurrence of AIS.

Furthermore, molecular docking studies showed that important targets such as AKT1, BAD, PIK3CG, PRKG1, MAPK1, and PDE5A have good binding affinity for ICS II, and MD simulations confirmed the stability of the interaction between important target (PRKG1) and ICS II.

## Conclusion

In summary, the practice of ICS II for AIS has demonstrated its efficacy and benefits. ICS II is promising as an alternative drug to the representative PDE5A inhibitor (sildenafil) for the treatment of AIS. The cGMP-PKG signaling pathway and ICS II are very promising strategies for the treatment of AIS. Basic studies, network pharmacology, molecular docking, and MD simulations confirm that ICS II attenuates AIS injury through multiple pathways such as anti-inflammatory, anti-apoptotic and anti-oxidative stress. However, the current understanding of the molecular mechanism of ICS II is mainly derived from network pharmacology, in vivo animal experiments and in vitro cellular studies. Preclinical AIS models do not necessarily represent the pathophysiology of human AIS, in part because laboratory animals are typically young men without underlying disease. In addition, the approach selected for ICS II in many studies was pretreatment before manufacturing AIS animal disease models. This is an unlikely treatment option for AIS in humans because the onset of stroke is unpredictable. Therefore, rationally designed in vitro and in vivo studies based on these speculations are needed to validate the predicted mechanisms. Thus, the aforementioned limitations of ICS II provide opportunities for further expansion and in-depth studies of its clinical efficacy validation and pharmacological mechanisms of action.

## Supplementary Information

**Additional file 1: Supplementary file 1.** PRISMA Checklist.

**Additional file 2: Supplementary file 2.** PubMed’s keyword search strings.

**Additional file 3.** Quality assessment of CAMARADES and ARRIVE.

**Additional file 4.** Information of AIS and ICS II targets.

## Data Availability

All data in this study are included in this article and its supplementary information files.

## Abbreviations

AIS: Acute ischemic stroke
ARRIVE: Animal Research: Reporting in Vivo Experiments
BBB: Blood–Brain Barrier
BCCAO: Bilateral common carotid artery occlusion
BP: Biological process
CC: Cellular component
CNKI: Chinese National Knowledge Infrastructure
DALYs: Disability adjusted life years
DEGs: The differentially expressed genes
GEO: Gene Expression Omnibus
GO: Gene ontology
ICS II: Icariside II
KEGG: Kyoto Encyclopedia of Genes and Genomes
MCAO: Middle cerebral artery occlusionl
MD: Molecular dynamics
MF: Molecular function
PDE5i: Phosphodiesterase 5 inhibitors
ROS: Reactive oxygen species
SOD: Superoxide dismutase
VIP: Chinese Science and Technology Periodicals Database
