# Supplementary material for: Pharmacological mechanism and therapeutic efficacy of Icariside II in the treatment of acute ischemic stroke: a systematic review and network pharmacological analysis
Source: BMC Complement Med Ther. 2022 Sep 30;22:253. doi: 10.1186/s12906-022-03732-9 (PMC9526298; doi:10.1186/s12906-022-03732-9)
Supplement: Supplementary file 2 — Additional file 2: Supplementary file 2. PubMed’s keyword search strings. [file 12906_2022_3732_MOESM2_ESM.pdf]

Supplementary file2: PubMed's keyword search strings.

((((((((((((((((((((((((Ischemic Strokes) OR (Stroke, Ischemic)) OR (Ischaemic Stroke)) OR (Ischaemic Strokes)) OR (Stroke, Ischaemic)) OR (Cryptogenic Ischemic Stroke)) OR (Cryptogenic Ischemic Strokes)) OR (Cryptogenic Ischemic Strokes)) OR (Stroke, Cryptogenic Ischemic)) OR (Cryptogenic Stroke)) OR (Cryptogenic Strokes)) OR (Stroke, Cryptogenic)) OR (Cryptogenic Embolism Stroke)) OR (Cryptogenic Embolism Strokes)) OR (Embolism Stroke, Cryptogenic)) OR (Stroke, Cryptogenic Embolism)) OR (Wake-up Stroke)) OR (Stroke, Wake-up)) OR (Wake up Stroke)) OR (Wake up Stroke)) OR (Acute Ischemic Stroke)) OR (Acute Ischemic Stroke)) OR (Ischemic Stroke, Acute)) OR (Stroke, Acute Ischemic)) AND (((("baohuoside I" [Supplementary Concept]) OR (icarisid II)) OR (icariside II)) OR (3,5,7-trihydroxy-4'-methoxyl-8-prenylflavone-3-O-rhamnopyranoside))
